# Supplementary figures and images for: Data-driven predictions of potential Leishmania vectors in the Americas
Source: PLoS Negl Trop Dis. 2023 Feb 21;17(2):e0010749. doi: 10.1371/journal.pntd.0010749 (PMC9983874; doi:10.1371/journal.pntd.0010749)

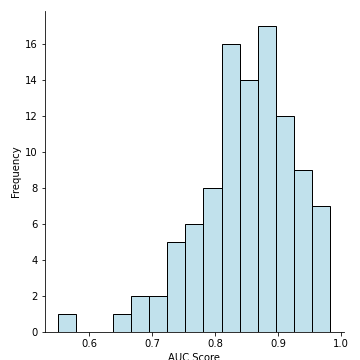

Supplement: S1 Fig — The average AUC score was 0.851, and the median AUC score was 0.863. An AUC = 1.0 means the model is perfectly able to distinguish between the sandflies that are vectors and those that are not. (TIFF) [file pntd.0010749.s007.tiff]

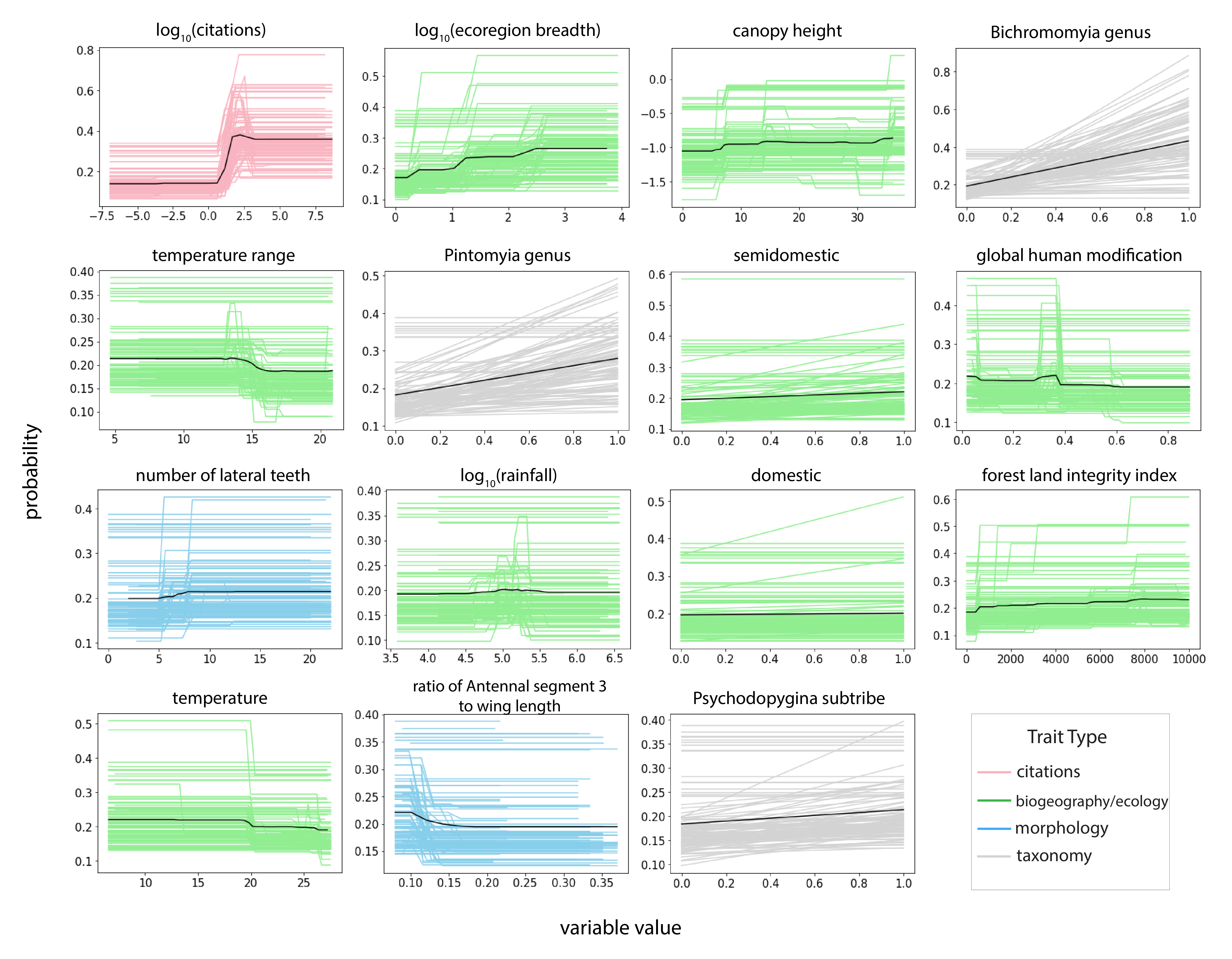

Supplement: S2 Fig — The trait value is shown on the x-axis, and the importance is shown on the y-axis. Colored lines represent the marginal dependence of the trait from the 100 BRT models, while the solid black line represents the average dependence. The definition of each variable can be found in S1 Table. (TIFF) [file pntd.0010749.s008.tiff]

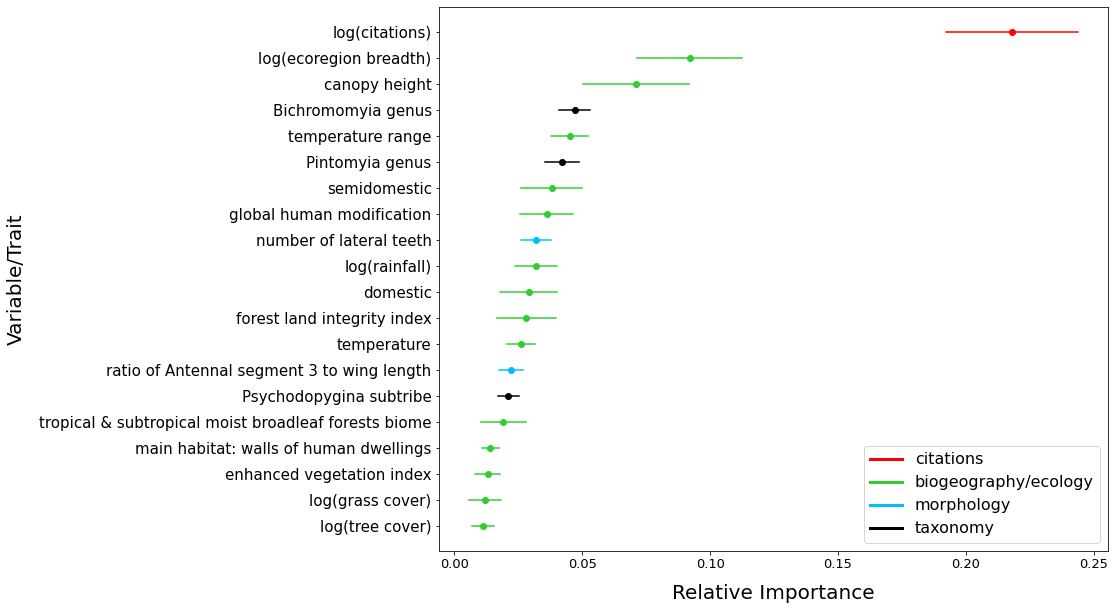

Supplement: S3 Fig — Points represent mean gain value across 100 iterations and error bars represent 95% bootstrapped confidence intervals. Categorial variables are not summed here; each variable is left as it’s own. (TIFF) [file pntd.0010749.s009.tiff]

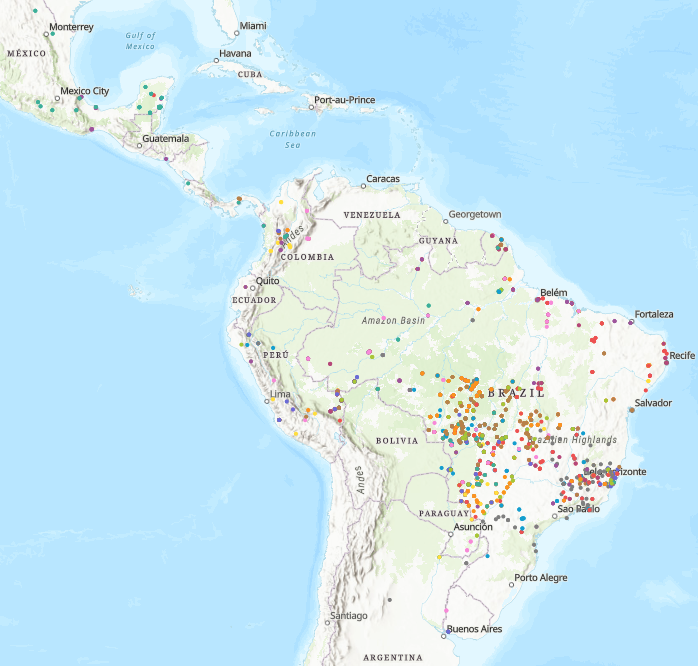

Supplement: S4 Fig — (TIFF) [file pntd.0010749.s010.tiff]

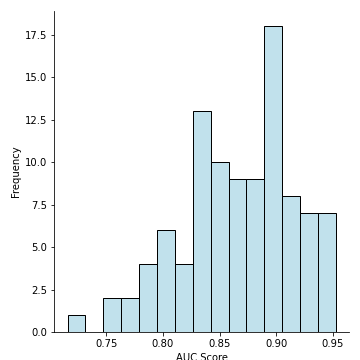

Supplement: S6 Fig — The average AUC score was 0.867, and the median AUC score was 0.869. (TIFF) [file pntd.0010749.s012.tiff]

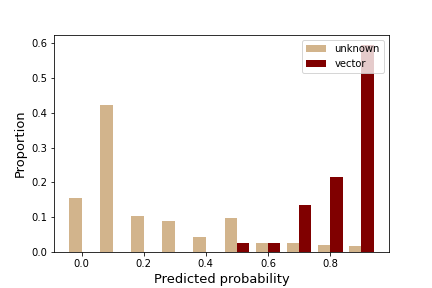

Supplement: S7 Fig — Red bars indicate the proportion of confirmed vectors that were predicted at that probability, while beige bars indicate the proportion of non-vector sandflies that were predicted at that probability. (TIFF) [file pntd.0010749.s013.tiff]

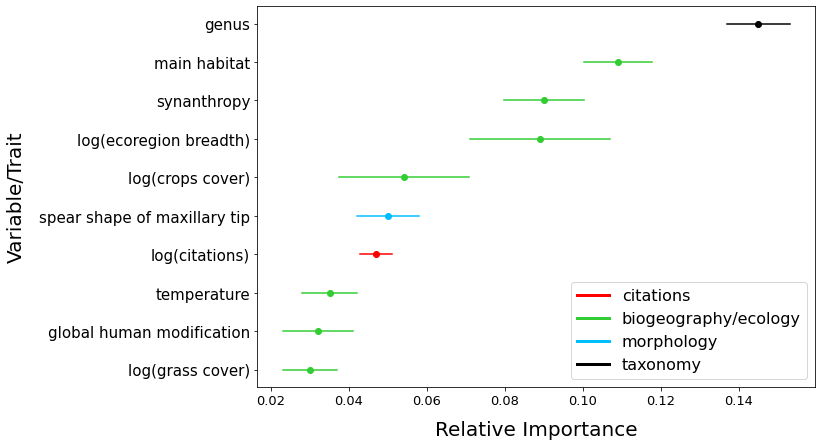

Supplement: S8 Fig — Points represent mean gain value across 100 iterations. The importances for binary variables were summed up to obtain a single value for the entire categorical variable. (TIFF) [file pntd.0010749.s014.tiff]

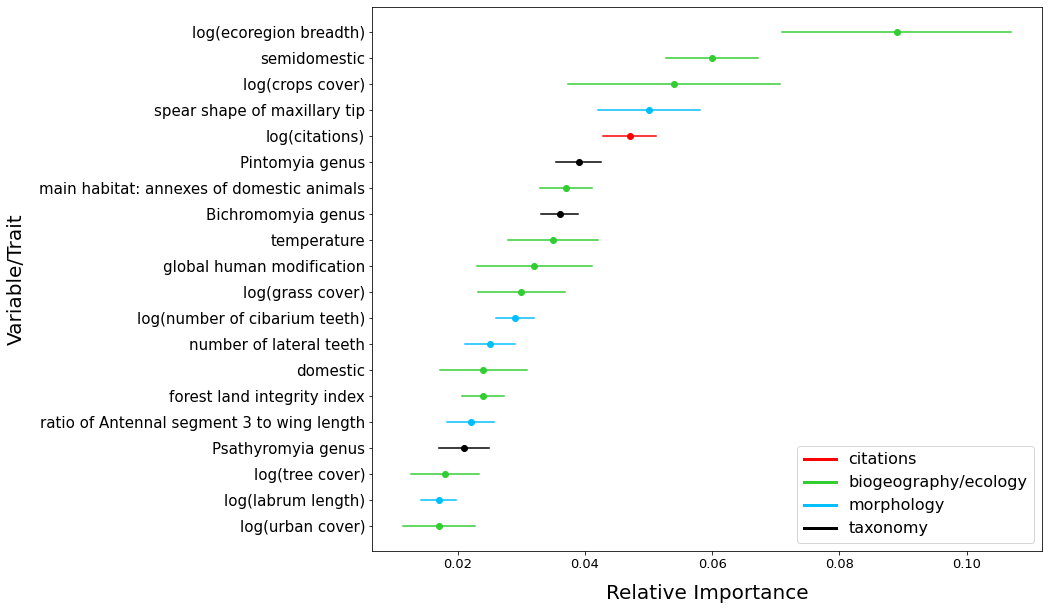

Supplement: S9 Fig — Points represent mean gain value across 100 iterations and error bars represent 95% bootstrapped confidence intervals. Categorial variables are not summed here; each variable is left as its own. (TIFF) [file pntd.0010749.s015.tiff]

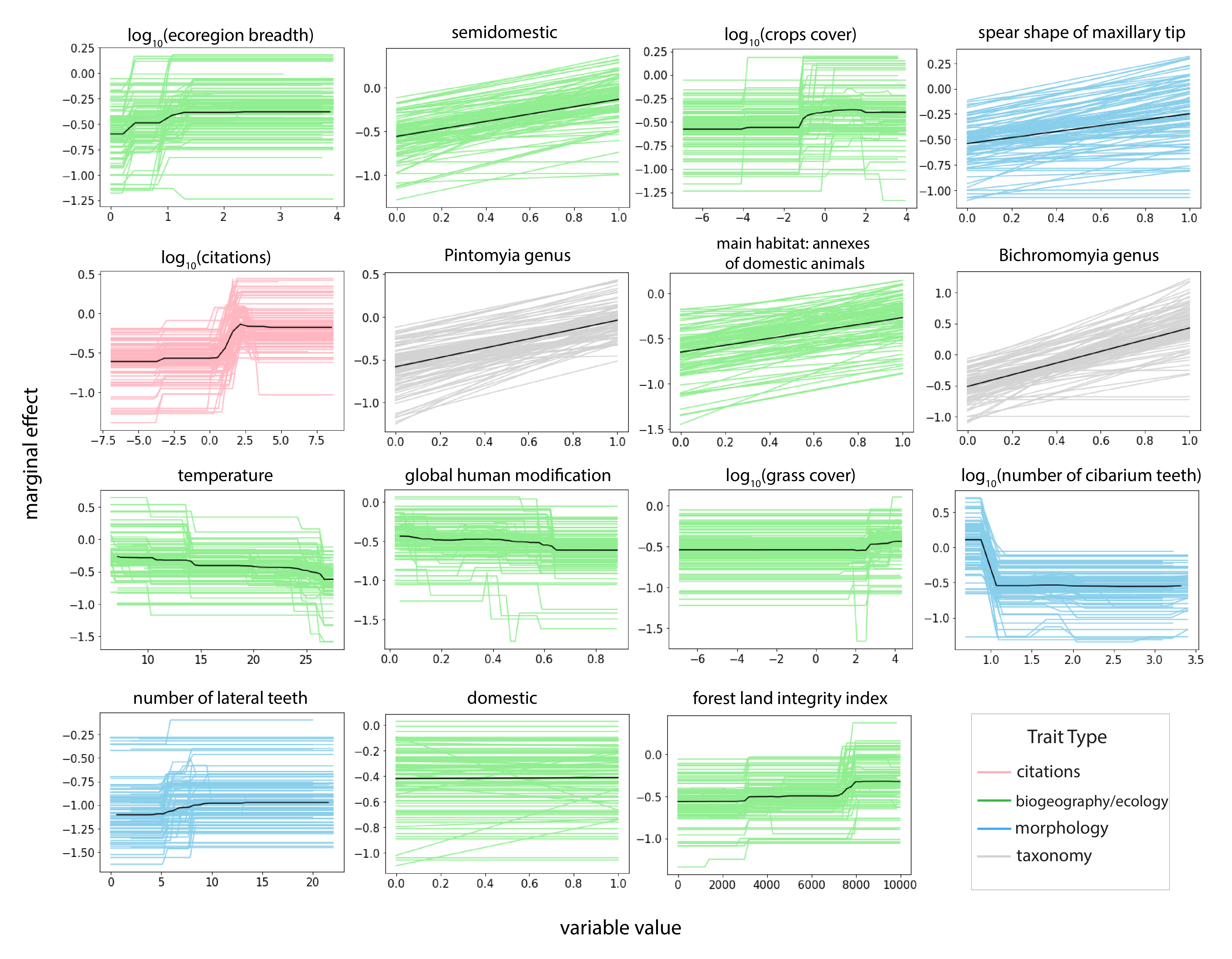

Supplement: S10 Fig — Variable value is shown on the x-axis, and marginal effect is shown on the y-axis. Partial dependence plots show the dependence of the probability on that trait’s value, i.e., how the vector probability changes as the trait value increases. (TIFF) [file pntd.0010749.s016.tiff]
