# Supplementary material for: Data-driven predictions of potential Leishmania vectors in the Americas
Source: PLoS Negl Trop Dis. 2023 Feb 21;17(2):e0010749. doi: 10.1371/journal.pntd.0010749 (PMC9983874; doi:10.1371/journal.pntd.0010749)
Supplement: Attachment — Submitted filename: Review Comments.docx [file pntd.0010749.s017.docx]

**Revisions for:** Data-Driven Predictions of Potential Leishmania Vectors in Latin America

**Editor comments:**

Reviewer #1: This is a very interesting paper and must be published.

Reviewer #2: The authors here combine data compilation and machine learning to predict likely but currently unknown sandfly vectors of Leishmania. The work is generally sound, but I did have some major comments regarding (i) the geographic and taxonomic scope of the sandfly species considered (and broadening to the Americas as a whole), possible inclusion of other evolutionary features, and some details regarding the modeling methods itself.

1. L14 and elsewhere: There are a few instances across the manuscript when parasites/diseases are conflated (e.g., “a parasitic disease transmitted by… on L14 and L34, “disease transmission” on L37, etc). The authors largely do a nice job referring to parasite transmission (given that parasites are transmitted between vectors and hosts, rather than the clinical diseases they cause), but a few additional cases could be resolved.

Response: The phrases mentioned by the reviewer now differentiate between parasite transmission and disease occurrence.

*Updated:*

*L 15: In particular, incidence of American Cutaneous Leishmaniasis (ACL), a disease caused by parasites transmitted by sandflies, is increasing as previously intact habitats are cleared for agriculture and urban areas, potentially bringing people into contact with vectors and reservoir hosts.*

*L 34: American Cutaneous Leishmaniasis (ACL) is a neglected disease caused by sandfly-transmitted parasites in the Americas. There is an incomplete understanding of which sandfly species transmit the parasite, complicating efforts to limit parasite transmission and consequently, disease burden. In this study, the authors created a database of sandfly traits, then used predictive models to determine important factors in parasite transmission and how different climate and environmental variables predict which vectors can transmit the parasites that cause ACL.*

*L 41: This new knowledge can lead to a better understanding of the system of transmission, and can point to possible hotspots of risk. The analysis can also help direct researchers to areas of interest for sampling studies, as well as specific sandflies on which to focus effort.*

*L 45: American cutaneous leishmaniasis (ACL) is a neglected tropical disease caused by parasites in the genus Leishmania, and transmitted by sandflies of the subfamily Phlebotominae [1, 2].*

*L 77: Climate change, deforestation, travel, and natural disasters are correlated with the spread of the parasite and leishmaniasis [2], yet it is hard to mechanistically predict the effect of global change on leishmaniasis incidence and distribution due to gaps in understanding of the parasite transmission cycle.*

*L 83: However, temperature responses likely vary by sandfly species [14, 15], and thus the effect of climate warming on Leishmania transmission cannot be predicted precisely as the full range of sandflies that could transmit the parasites have yet to be described.*

2. L46 and L109: Clarify that the species being considered are exclusively in the subfamily Phlebotominae.

Response: The species are clarified as being exclusively Phlebotomine sandflies.

*Updated: L 46: American cutaneous leishmaniasis (ACL) is a neglected tropical disease caused by parasites in the genus Leishmania, and spread by sandflies of the subfamily Phlebotominae [1, 2].*

*L 109: We first compiled a database on Phlebotomine sandfly vector status as well as behavioral, morphological, taxonomic, and ecological traits that could be used to delineate vectors from non-vectors.*

3. L53: The authors mention expansion of ACL cases into the southern US, but the models seem restricted to Central/South America. Are all sandfly species in North America also present in Central/South America? The taxonomic scale of the models is somewhat unclear.

Response: The sandfly species that have observations in North America are included in our database. We went back to the plots and found a few occurrence points of known vectors in North America, after which we updated the maps accordingly. These points have already been included in the computational analysis; they were only missing from the map figures. We have changed the title of the paper to reflect that the scope of these data points extends to all of the Americas, rather than only Central and South America. We appreciate this helpful point.

*L 1: Data-Driven Predictions of Potential Leishmania Vectors in the Americas*

*L 34: American Cutaneous Leishmaniasis (ACL) is a neglected disease caused by sandfly-transmitted parasites in the Americas.*

*L 55: Similar to other disease-causing parasites, Leishmania parasites are found in wild and domestic reservoir hosts, which are located across the Americas, and are picked up by sandfly females during their blood meal before laying eggs [2, 8, 9].*

*L 318: Our primary model leveraged ecological, behavioral, and biogeographic characteristics of sandfly species found across the Americas to predict the probability of a sandfly being a vector of ACL.*

*L 326: Study effort may indicate that Leishmania vectors are undersampled, but it may also indicate that vectors have a broad range that interfaces with human settlements, as it would be sampled in many different field surveys across the Americas.*

*L 407: Instead, we opted for a genus-wide model that includes all Leishmania parasites that cause cutaneous leishmaniasis in the Americas.*

*L 419: Additionally, although North American sandflies were included in the models, there were few occurrence points for these species, and they did not have high vector prediction scores. This is another limitation of our analysis considering the rise in North American cases of leishmaniasis and potential range shifts of sandfly vectors.*

4. L109 and L125: Are the 512 sandfly species those restricted to the Americas as a whole or to only Central and South America?

Response: The 512 sandflies are not restricted to only Central and South America (see comment 3). We have clarified accordingly.

*L 125: Our dataset comprised 512 documented sandfly species across the Americas (sample size n = 512).*

5. L167: The authors mention genus here, but this is not mentioned in the earlier methods text on which traits were obtained. I also think the authors have missed an opportunity to also include evolutionary features of the sand fly species. Could the authors consider quantitative information (e.g., evolutionary isolation, phylogenetic diversity) from a sandfly phylogeny? Several other recent BRT-style papers have found such traits to be important.

Response: We agree with the reviewer that phylogeny is important, but there is unfortunately not enough quantitative data about sandfly phylogeny currently available; specifically, there is not a complete phylogeny that can be used to calculate an evolutionary distance among the sandflies. Regarding the genus trait in the models, we separated the sandflies by genus, making each genus a binary trait. After training the models and getting variable importance for each individual genus, we used the sum of these variable importances to get the importance of the genus trait overall (Evans et al. 2017). We have added a sentence in the methods and discussion about this.

*Updated L 131: We included genus as a variable to include a measure of taxonomic relatedness in the model.*

*L 211: For the variables we converted from categorical to binary, the relative importance of each binary trait was summed to represent the importance for the overall categorical variable.*

*L 318: Our primary model leveraged ecological, behavioral, taxonomic, and biogeographic characteristics of sandfly species found across the Americas to predict the probability of a sandfly being a vector of ACL.*

*L 349: This suggests certain traits that occur in these parts of the sandfly phylogenetic tree are important for vector status, but full sandfly phylogenies were unavailable, so we were unable to quantitatively asses the effect of genetic distance. This highlights the importance of careful taxonomic work as well as high quality genetic data for as many species as possible.*

6. L161: A 10% feature coverage threshold seems rather low. Could the authors include a histogram of trait coverage across the 512 species? It would be helpful to see how coverage breaks down (e.g., if most of your features have very high coverage) and adjust your threshold to a higher cutoff (e.g., 50%), which should also improve your ability to predict novel vectors (if important traits have poor coverage).

Response: The 10% is arbitrary to some extent, but has worked well in the past for filtering out variables with low coverage to simplify the models a bit. Increasing or decreasing this number does not make a large difference in model performance or training importance. In order to check whether the cutoff changes the predictions, we conducted a cutoff analysis where we ran the model with no traits removed, and another time with a 20% trait coverage cutoff (i.e., traits were only included if >= 20% of the species had data for the trait). For both of these models, the top predictions are nearly identical to the original model that included traits with 10% coverage in model predictions, and the variable importances are also nearly identical. In theory, a data coverage cutoff is not necessary because of the way boosted regression treats missing data - it views the missingness as a 'common value' to group on. This is why, for example, if you have nonrandom patterns of missingness (e.g., all of the understudied species are missing values for a certain trait) the algorithm captures its effect, to some degree. If one were to include all of the data regardless of coverage, low coverage variables that also have "low information" (either due to low coverage or due to the feature not being consequential for prediction) have low relative importance scores. Co-author and machine learning expert Barbara Han has used the 10% cutoff previously in her papers (Evans et al. 2017, Han et al. 2019, Fischhoff et al. 2021, Han et al. 2015). We also include an additional column in the supplementary trait table with the coverage percentage of each trait.

*Line 161: Following (Evans et al. 2017, Han et al. 2019, Fischhoff et al. 2021, Han et al. 2015), variables with less than 10 percent coverage and a correlation factor greater than 0.7 with other variables were not included in the final analysis to avoid overfitting and misestimating the importance of highly correlated variables. The cutoff removes traits for which less than 10 percent of sandflies have data, to filter out variables with low coverage to simplify the models a bit. In theory, a data coverage cutoff is not necessary because of the way boosted regression treats missing data - it views the missingness as a 'common value' to group on. If one were to include all of the data regardless of coverage, low coverage variables that also have "low information" (either due to low coverage or due to the feature not being consequential for prediction) have low relative importance scores. As such, we used the 10% cutoff to simplify the model by removing variables that are likely to not contribute to model performance and predictions due to "low information".*

7. L189: When splitting data into training and test sets, did the authors perform stratified sampling via the positive labels? This would be important to ensure that the same fraction of positive labels are in both training and test splits, rather than one set having more proportional positives by random.

Response: Yes, we did perform stratification via the positive labels to ensure proportional splitting in the training and testing sets. We updated the text to clarify this point.

*Updated 189: For training and tuning analysis, the data was stratified and split into 80% training and 20% testing sets such that each set had an equal proportion of positive labels.*

8. L199: Please include final parameter selections for the models.

Response: For both our models (primary and secondary), we generate an ensemble of 100 models from 10 different sets of parameters that we have tuned to the data. We feel that it would be misleading to include final parameter selections since there are 20 total parameter sets (10 for the primary model and 10 for the secondary one). However, we have a github repository with the provided parameters and all the data for repeatability. We have clarified this in the text.

*Updated L 199: We use the 10 best performing parameter sets (minimum log-loss) in our BRT models.*

9. L232: If the authors are going to refer to the models as BRTs, I would use this earlier in the methods (e.g., within L171-179).

Response: We agree with this comment.

*Updated L 171: We used extreme boosting through the XGBoost library in Python to fit a logistic classifier boosted regression tree model (BRT).*

10. L235: Can you provide not just the median but also a measure of variance (e.g., SE)?

Response: We agree with this comment.

*Updated L 235: The models achieved a high aggregate median out-of-sample AUC of 0.86 with a standard error of 0.008 across the 100 model iterations (Figure S1); therefore, on average, our models classified 86% of our observations correctly.*

11. L238 and L244-247: Because the authors use a 0.5 cutoff for predicted probability, the number of predicted-but-unknown vectors seems quite low. I would replace “some species” with “relatively few species” in the Figure 2 legend. The authors could keep the 50% cutoff here or consider instead using a 95% sensitivity threshold (e.g., Mull et al. 2022, Carlson et al. 2022).

Response: We choose a threshold of 0.5 as this is a conservative estimate of the probability that separates the 1s (positive values = known vectors) from the 0s (negative values = unknown vectors). Further, 0.5 is a standard probability for separating positive values and negative values when examining model performance through sensitivity, specificity, and AUC (a aggregate measure of sensitivity and specificity). We did consider a 95% threshold, but decided that 0.5 is more robust, and because of data sparsity, it is likely that we are missing species that might be vectors. The secondary analysis provides additional species that might be of consequence as well.

*Updated L 238: Figure 2: The model accurately classifies known vectors and identifies relatively few species of unknown status as likely vectors.*

12. L268: One limitation of the maps here is that it is impossible to infer the density of different species. Could the authors consider also overlaying geographic distribution shapefiles of known/unknown vectors? Or coloring points by their corresponding vector species? The authors should also clarify from where these data are derived (e.g., GBIF?). Figure 3 also suggests the authors limit their data into northern Mexico, but I do wonder if expanding your dataset into sandflies present in North America would be interesting/worthwhile (especially giving some of the earlier framing in the Introduction about detecting range shifts of vectors with climate change).

Response: See comment 3 and 4. Because sandflies are restricted to specific habitats and can only fly 30-300 meters, we felt that it was more precise to use occurrence points than shapefiles. We have updated the map figures in the main text with the North American points that were not plotted previously, and added supplementary maps (Figure S4, Figure S5) that show species richness/density by coloring points by sandfly species.

*Updated L 267: [updated maps]*

*L 286: Figure 3: Confirmed (A) and newly-predicted (B) vectors occur throughout the Americas. (A) Observed occurrences of confirmed vectors of Leishmania spp. that cause ACL, taken from GBIF and plotted in arcGIS [81, 82]. (B) Observed occurrences of sandflies of unknown vector status that our models assigned a predicted probability above 0.5. Most predicted vectors are in Brazil due to more extensive survey efforts and availability of public data [81, 82]. Maps showing species richness and vector distribution for each species of Leishmania spp. can be found in the supplementary materials (Figure S4, Figure S5).*

13. L285 and L296: I think it’d be worth reminding the readers what differentiated the response variable in the primary and secondary models.

Response: We agree with this comment.

*Updated L185: The four most important features in our primary model (i.e. trained on confirmed vectors) are (i) the number of citations in PubMed, (ii) the genus of the sandfly, (iii) number of ecoregions the sandfly inhabits, and (iv) mean canopy height.*

*Updated L296: The top features of our secondary model (i.e. trained on confirmed and potential vectors) were (i) the genus of the sandfly, (ii) the sandfly’s main habitat, (iii) synanthropy, and (iv) number of ecoregions the sandfly inhabits (Table S6); the full list can be found in the Supplementary materials.*

14. L319: Why not extent the species pool to those in North America (i.e., all Americas), especially given the earlier framing about climate shifts?

Response: See comment 3, 4, and 12. We corrected the error in our maps, which now show the North American sandfly species. We also updated places in the text to clarify that the range of our study encompasses all of the Americas.

15. L320: I would suggest “identify several previously unknown species with a relatively high probability…”, to temper language here.

Response: We agree with this comment.

*Updated L 321: The group of 100 BRT models was able to classify sandfly vectors with 86% accuracy and identified several previously unknown species with a relatively high probability of being a vector.*
